# Detection Study of Bipolar Depression Through the Application of a Model-Based Algorithm in Terms of Clinical Feature and Peripheral Biomarkers

**DOI:** 10.3389/fpsyt.2019.00266

**Published:** 2019-05-01

**Authors:** Yanqun Zheng, Shen He, Tianhong Zhang, Zhiguang Lin, Shenxun Shi, Yiru Fang, Kaida Jiang, Xiaohua Liu

**Affiliations:** ^1^Department of Psychiatry, Shanghai Mental Health Center, Shanghai Jiao Tong University School of Medicine, Shanghai, China; ^2^Shanghai Key Laboratory of Psychotic Disorders, Shanghai Mental Health Center, Shanghai Jiao Tong University School of Medicine, Shanghai, China; ^3^Biochemistry Laboratory, Shanghai Mental Health Center, Shanghai Jiao Tong University School of Medicine, Shanghai, China; ^4^Department of Psychiatry, Huashan Hospital affiliated to Fudan University, Shanghai, China

**Keywords:** bipolar depression, model-based algorithm, neurotrophic factor, clinical feature, biomarker

## Abstract

**Objectives:** The nature of the diagnostic classification of mood disorder is a typical dichotomous data problem and the method of combining different dimensions of evidences to make judgments might be more statistically reliable. In this paper, we aimed to explore whether peripheral neurotrophic factors could be helpful for early detection of bipolar depression.

**Methods:** A screening method combining peripheral biomarkers and clinical characteristics was applied in 30 patients with major depressive disorder (MDD) and 23 patients with depressive episode of bipolar disorder. By a model-based algorithm, some information was extracted from the dataset and used as a “model” to approach penalized regression model for stably differential diagnosis for bipolar depression.

**Results:** A simple and efficient model of approaching the diagnosis of individuals with depressive symptoms was established with a fitting degree (90.58%) and an acceptable cross-validation error rate. Neurotrophic factors of our interest were successfully screened out from the feature selection and optimized model performance as reliable predictive variables.

**Conclusion:** It seems to be feasible to combine different types of clinical characteristics with biomarkers in order to detect bipolarity of all depressive episodes. Neurotrophic factors of our interest presented its stable discriminant potentiality in unipolar and bipolar depression, deserving validation analysis in larger samples.

## Introduction

The *Diagnostic and Statistical Manual of Mental Disorders, Fifth Edition* (*DSM-5*) separates the mood disorders into two sections: depressive and related disorders, and bipolar and related disorders. The new version of the diagnostic manual of bipolar disorders emphasizes more specific manifestations related to hypomanic and mixed manic states, which are considered to be a separate class of mood disorder “in terms of symptomatology, family history and genetics” (1–3). From a statistical point of view, the nature of the diagnostic classification of depressive disorder can be clearly taken as a typical dichotomous problem with only two possible outcomes: one is bipolar depressive disorder (BPD) and the other is major depressive disorder (MDD).

In spite of its clear-cut division in diagnostic manual, a definite diagnosis for bipolar disorder remains an elusive goal. In a 10-year follow-up study of 290 unipolar depressed patients, Holmskov et al. have reported that the overall risk of conversion from initial diagnosis of unipolar depression to later bipolar disorder reaches up to 20.7% (4). The major challenge for clinical decision is that the identification of psychopathology still relies on the clinician’s subjective judgment. Generally speaking, the classification of mood disorder or the diagnosis of bipolar disorder can be made without difficulty with a manic episode. But in the absence of specific symptoms, the clinical decision is hard to be made, especially for those patients with bipolar depression who initially come for medical help as depressive or other nonspecific symptoms being fairly laid open to doctor.

The search for objective or subjective assessment of whether a depressive episode is potentially subordinate to bipolar disorder or not is of clinical relevance, since patients at high risk may be missing the optimized opportunity of therapy. There have been several clinical studies that focused on relevant risk factors of bipolar disorder in terms of clinical symptoms for early detection. In the study mentioned above, Holmskov et al. have performed analysis for the risk factor for conversion at baseline: a rising number of previous depression recurrences [hazard ratio (HR) 1.18, 95% confidence interval (CI) (1.10–1.26)] and no strong relationship between gender, age at onset, subtype of depression, and any of the investigated Hamilton Depression Scale (HAM-D) subscales with the conversion, among others (4). It was said that it would take an average of 10 years for misdiagnosed patients to get the right diagnosis and treatment of BD (5). As for the children and youth cohort, a systematic review of cross-sectional studies reported that pediatric patients with bipolar depression had higher levels of depression severity, psychiatric comorbidity, and family history (6).

Using clinical characteristics alone is not a precise and stable solution for identification of bipolar disorder. It is prone to a certain probability of misjudgment when clinicians use individual or several indicators for clinical classification. In view of the overlap of clinical manifestations of unipolar and bipolar disorder as well as the limitations of clinician’s subjective experience, methods integrating different dimensions of evidences to make judgments might be more statistically reliable and sufficient than independent variables. The scientific research has made some progress in searching for biomarkers being objectively indicative of mood disorders. Chang et al. have found that C-reactive protein could be a differential biomarker making out bipolar II depression versus MDD (7), although it was challenged by another study as confounding factors in a case–control study (8). Morphometric analyses using voxel-based morphometry by Redlich et al. have demonstrated that structural abnormalities in neural regions supporting emotion processing, such as gray matter volumes in the hippocampus and amygdala and white matter volumes within the cerebellum and hippocampus, could be good markers (9). The pattern classification approach was announced, reaching up to 79% accuracy, but the model did not survive the Alpha-Sim correction in validation data (false-positive rate is too high when applied in test data).

In our previous work, we have found that levels and trends of serum neurotrophic factors differed between patients with unipolar and bipolar depression, which may give us some inspiration. Factors such as fibroblast growth factor (FGF)-2, vascular endothelial growth factor (VEGF), nerve growth factor (NGF), and insulin-like growth factor (IGF)-1 might be potential candidate biomarkers for bipolar disorder. Drug-naïve patients with bipolar disorder with manic episode showed increased serum levels of FGF-2, NGF, and IGF-1, while patients with MDD showed decreased serum FGF-2 levels that are probably associated with their compensatory roles of neuroprotection and angiogenesis, which are involved in their specific pathophysiology in these two disorders and thus be able to differentiate from each other (10, 11). To our knowledge, their clinical application for diagnostic assistance of mood disorders remains uncertain so far, although these neurotrophic factors potentially could be robust and biologically interpretable biomarkers.

In this study, we present a correlation-based feature selection and a reliability-based optimization strategy to extract enough information from unipolar depression and bipolar depression samples. Here, not only would we aim to investigate whether and to what extent neurotrophic factors and their individual components can be related to either unipolar or bipolar depression, we would also try to establish simple artificial intelligence system for stably differential diagnoses for bipolar depression by combining biological biomarkers and clinical characteristics. To our knowledge, there are few similar studies so far. We hypothesized that peripheral blood biomarkers can be successfully screened out from the feature selection and optimize model performance as reliable predictive variables.

## Materials and Methods

### Subjects, Blood Sample Collection, and Laboratory Test

Patients in a depressive episode, including 30 patients with MDD and 23 patients with BPD, were recruited in Shanghai Mental Health Center, Shanghai Jiao Tong University School of Medicine in 2014. The inclusion criteria were as follows: 1) age 18–60 years; 2) met the criteria of the *Diagnostic and Statistical Manual of Mental Disorders*, *4th edition* (*DSM-IV*, 12) for major depressive episode and depressive episode of bipolar disorder; and 3) patients not taking any psychiatric medications at least 2 weeks before treatment. Patients with severe physical illness and other mental illness associated with depressive state were excluded from this study. Subjects who are currently pregnant or lactating were also excluded.

The demographic information was collected during enrollment. The 24-item Hamilton Depression Scale (HAMD-24), the Montgomery–Åsberg Depression Rating Scale (MADRS), and the Hamilton Anxiety Scale (HAMA) were measured to assess the clinical symptoms of patients. The neurotrophic factors (FGF-2, NGF, IGF-1, and VEGF) in peripheral blood of all patients with MDD and BPD were measured by enzyme-linked immunosorbent assay (ELISA) technique. All patients received 8 weeks of personalized therapy, among which 10% MDD patients and 60% of BPD patients took mood stabilizers. Both clinical symptom assessment and blood test took place at baseline and after treatment. Detailed information was published in our previous paper (10, 11).

The study protocol was approved by the Ethics Committee of Shanghai Mental Health Center, Shanghai Jiao Tong University School of Medicine. All subjects provided informed consent for this study.

### Statistical Analysis and Model-Based Diagnostic Algorithm

Patients’ characteristics of a three-dimensional dataset containing peripheral levels of neurotrophic factors, clinical scale scores, and demographic features (as shown in **Table 1**) were used for feature selection, of which discriminatory power was evaluated stepwise and search strategy was approached for global optima. Then, a model-based algorithm was applied to reduce the dimension and boost model performance. Identification of robust biomarkers and model performance was supervised by significant level of analysis of covariance and size effect as well as error rates based on cross-validation.

**Table 1:** Variables that belonged to the “three-dimensional dataset” for feature selection.

Variable	Variable no./Symptom code	Additional information/Composed item
Sociodemographic and clinical characteristics		
Age (years)	age1	
Age at onset (years)	age2	
Presence of psychotic symptoms	Psycho_categor	
Presence of family history	History_categor	
HAMD-24		
Factor 1: Anxiety/Somatization		
Baseline	dfactor1b	Item 10 score of the HAMD Scale (Anxiety-Psychic)
Delta effect	ddfactor1	Item 11 score of the HAMD Scale (Anxiety-Somatic)
		Item 12 score of the HAMD Scale (Somatic symptoms-gastrointestinal)
		Item 15 score of the HAMD Scale (Hypochondriasis)
		Item 17 score of the HAMD Scale (Insight)
Factor 2: Weight loss		
Baseline	dfactor2b	Item 16 score of the HAMD Scale (Loss of weight)
Delta effect	ddfactor2	
Factor 3: Cognitive dysfunction		
Baseline	dfactor3b	Item 2 score of the HAMD Scale (Feeling of guilt)
Delta effect	ddfactor3	Item 3 score of the HAMD Scale (Suicide)
		Item 9 score of the HAMD Scale (Agitation)
Factor 4: Diurnal variation		
Baseline	dfactor4b	Item 18 score of the HAMD Scale (Diurnal variation)
Delta effect	ddfactor4	
Factor 5: Loss of motivated behavior		
Baseline	dfactor5b	Item 1 score of the HAMD Scale (Depressed mood)
Delta effect	ddfactor5	Item 7 score of the HAMD Scale (Work and interests)
		Item 8 score of the HAMD Scale (Retardation)
		Item 14 score of the HAMD Scale (Genital symptoms)
Factor 6: Sleep disturbance		
Baseline	dfactor6b	Item 4 score of the HAMD Scale (Insomnia-Initial)
Delta effect	ddfactor6	Item 5 score of the HAMD Scale (Insomnia-Middle)
		Item 6 score of the HAMD Scale (Insomnia-Delayed)
Factor 7: Despair/Sadness		
Baseline	dfactor7b	Item 22 score of the HAMD Scale (Sense of decline in ability)
Delta effect	ddfactor7	Item 23 score of the HAMD Scale (Feeling of despair)
		Item 24 score of the HAMD Scale (Feeling of inferiority)
MADRS		
Item 1 score of the MADRS Scale (Apparent sadness)		
Baseline	mads1b	
Delta effect	dmads1	
Item 2 score of the MADRS Scale (Reported sadness)		
Baseline	mads2b	
Delta effect	dmads2	
Item 3 score of the MADRS Scale (Inner tension)		
Baseline	mads3b	
Delta effect	dmads3	
Item 4 score of the MADRS Scale (Reduced sleep)		
Baseline	mads4b	
Delta effect	dmads4	
Item 5 score of the MADRS Scale (Loss of appetite)		
Baseline	mads5b	
Delta effect	dmads5	
Item 6 score of the MADRS Scale (Concentration difficulties)		
Baseline	mads6b	
Delta effect	dmads6	
Item 7 score of the MADRS Scale (Lassitude)		
Baseline	mads7b	
Delta effect	dmads7	
Item 8 score of the MADRS Scale (Inability to feel)		
Baseline	mads8b	
Delta effect	dmads8	
Item 9 score of the MADRS Scale (Pessimistic thoughts)		
Baseline	mads9b	
Delta effect	dmads9	
Item 10 score of the MADRS Scale (Suicidal thoughts)		
Baseline	mads10b	
Delta effect	dmads10	
HAMA		
Factor 1: Psychological anxiety factor		
Baseline	afactor1b	Item 1 score of the HAMA Scale (Anxious mood)
Delta effect	dafactor1	Item 2 score of the HAMA Scale (Tension)
		Item 3 score of the HAMA Scale (Fears)
		Item 4 score of the HAMA Scale (Insomnia)
		Item 5 score of the HAMA Scale (Intellectual)
		Item 6 score of the HAMA Scale (Depressed mood)
		Item 14 score of the HAMA Scale (Behavior at interview)
Factor 2: Somatic anxiety factor		
Baseline	afactor2b	Item 7 score of the HAMA Scale (Somatic-muscular)
Delta effect	dafactor2	Item 8 score of the HAMA Scale (Somatic-sensory)
		Item 9 score of the HAMA Scale (Cardiovascular symptoms)
		Item 10 score of the HAMA Scale (Respiratory symptoms)
		Item 11 score of the HAMA Scale (Gastrointestinal symptoms)
		Item 12 score of the HAMA Scale (Genitourinary symptoms)
		Item 13 score of the HAMA Scale (Autonomic symptoms)
FGF-2		
Baseline	FGF-2_B	
Delta for FGF-2	dFGF-2	
IGF-1		
Baseline	IGF_B	
Delta for IGF-1	dIGF	
VEGF		
Baseline	VEGF_B	
Delta for VEGF	dVEGF	
NGF		
Baseline	NGF_B	
Delta for NGF	dNGF	

HAMD-24, 24-item Hamilton Depression Scale; MADRS, Montgomery–Åsberg Depression Rating Scale; HAMA, Hamilton Anxiety Scale.

Statistical analyses were performed using SAS9.4 software for Windows (SAS Institute Inc., Cary, NC, USA). The demographic and clinical data of the two groups of patients were listed in the attached table (see **Supplementary Materials**), as were the serum neurotrophic levels we measured.

A stepwise discriminant analysis (method Forward Stepwise) was made to select variables for use in discriminating between the two groups, as measured by Wilks’ lambda, the likelihood ratio criterion (13). At each step, discriminant analysis evaluates all the variables and enters the one contributing most to the discriminatory power between groups. When none of the unselected variables meet the entry criterion, the forward selection process stops. Then, 11 variables in the dataset were found to have potential discriminatory power. Results of the selection process were summarized.

After that, the stepwise model was utilized for testing using the SAS Glmselect procedure. The “Glmselect” procedure, which is suitable for small sample research, has built-in penalties for model overfitting and internal collinearity of variables (14). Tenfold cross-validation was specified as a tuning method to choose an optimum model with minimum estimated prediction error (15). Multicollinearity was also a concern and was assessed by tolerance. Multiple logistic regression models were used to give maximum sensitivity and specificity as well as further analysis.

## Results

### Demographic and Clinical Characteristics

Of the 53 patients, 30 were MDD and 23 were BPD. The descriptive information on demographic and clinical characteristics is listed in **Table S1**. No significant difference in age and gender were found between the two groups. In our study, 79% of our patients were recurrent with an average age of 46 years old. Comparison of two groups showed that the duration of disorder (*Z* = 2.2559, *p* = 0.0241), number of previous episode (*Z* = 3.4131, *p* = 0.0006), and presence of family history (χ^2^ = 5.2170, *p* = 0.0308) were significantly higher in the BPD patients. Besides, there were no significant differences in the age, gender, educational level, marital status, age at onset, duration of the present episode, and the proportion of patients with psychotic symptoms between the two groups.

Also, no statistical differences were found between groups in the baseline HAMD Scale score, as well as the MADRS and the HAMA, as shown in **Table S2**. During the 8 weeks of follow-up, all patients finished a personalized therapy and 90.57% patients got a clinical remission with a reducing rate of Hamilton scale score ≥75% without group difference at the end. Additionally, we could not find any marked differences in the overall reducing rate between groups.

The serum levels of FGF-2, IGF-1, VEGF, and NGF in the two groups were shown as mean ± standard deviation (SD) in detail. No obvious differences between these four neurotrophic factors between groups were found at baseline and after treatment. However, the concentration trend of serum FGF-2 levels was completely different between groups (effect sizes = −2.118, *p* = 0.034); while MDD patients showed a distinct decline after treatment (*d* = 18.36 ± 94.06, *p* = 0.016), BPD patients maintained an insignificant change (*d* = −4.74 ± 92.58, *p* = 0.270) compared to the baseline level. No similar situation occurred in the other three neurotrophic factors. Notably, no correlation was shown between the serum FGF-2 concentration and the treatment wherein patients received a mood stabilizer or not (*Z* = 1.233, *p* = 0.218).

### Preliminary Screening of Predictive Variables by Discriminant Analysis

Stepwise discriminant analysis was conducted and the results are presented in **Table 2**. Since the test of homogeneity of within covariance matrices showed a significant χ^2^ value, the within covariance matrices were used in the discriminant function. Eleven variables in the dataset were found to have potential discriminatory power: 1) age at onset (years); 2) IGF-1 at baseline (ng/ml); 3) VEGF at baseline (pg/ml); 4) presence of family history; 5) presence of psychotic symptoms; 6) item 5 score of MADRS Scale (Loss of appetite); 7) HAMA Factor 1 at baseline (Psychological anxiety); 8) HAMD Factor 4 at baseline (Diurnal variation); 9) delta for FGF-2 (pg/ml); 10) delta for NGF (pg/ml); and 11) delta effect of HAMD Factor 2 (Weight loss). The total classification error rate of preliminary screening by discriminant analysis method was 0.2000 by re-substitution and was 0.3551 by cross-validation at this step. By cross-validation, only one patient (0.0435%) in BPD was misclassified into MDD while 20 patients (66.6667%) in MDD were misclassified into BPD. The results showed that the variables below together could simulate the patients in BPD well but held a high false-positive rate.

**Table 2:** Results of stepwise discriminant analysis.

Step	Variable	Partial *R*^2^	Pr > *F*	Average squared canonical correlation	Wilks’ Lambda
1	Presence of family history	0.0984	0.0222	0.0984	0.9016
2	Age at onset (years)	0.0652	0.0705	0.2344	0.7655
3	Presence of psychotic symptoms	0.0763	0.0521	0.2929	0.7070
4	Delta effect of HAMD Factor 2 (Weight loss)	0.0794	0.0524	0.4100	0.5900
5	Delta for FGF-2	0.0492	0.1340	0.4390	0.5610
6	IGF-1 at baseline	0.0536	0.1216	0.4691	0.5309
7	VEGF at baseline	0.0494	0.1374	0.4718	0.5282
8	Delta for NGF	0.1289	0.0154	0.5399	0.4601
9	HAMD Factor 4 at baseline (Diurnal variation)	0.1495	0.0087	0.6030	0.3970
10	The item 5 score of the MADRS Scale at baseline (Loss of appetite)	0.0689	0.0851	0.6304	0.3696
11	HAMA Factor 1 at baseline (Psychological anxiety)	0.0503	0.1480	0.6490	0.3510

### Dimensionality Reduction and Model Selection

**Figure 1:**
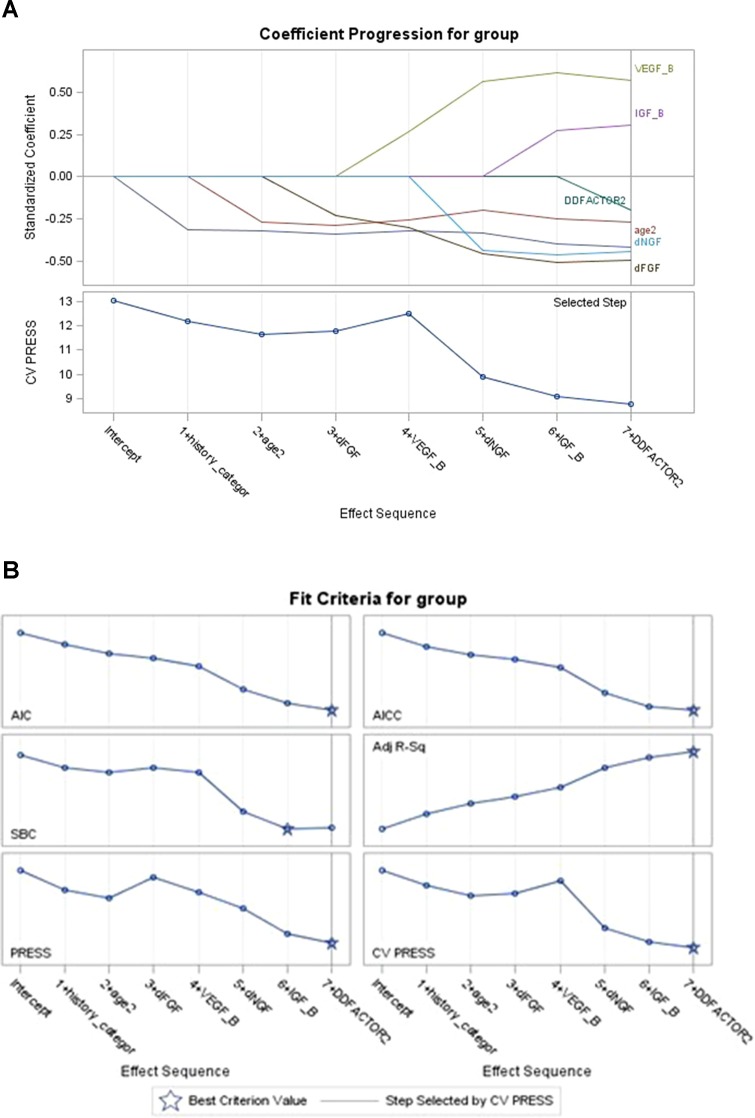
Coefficient of model selection procession **(A** and **B)**. The variables entered the model in turn (AIC criteria) while keeping the model false-positive rate steadily decreasing; “presence of family history” and “age at onset” in clinical data and “dFGF-2” in biomarkers data showed their best predictive effect for the outcome; “VEGF” slightly increased the cross-validation press of the model. Variables not shown in the figures mean that they met the cross-validation criterion in regularization step and had been dropped out (“diurnal mood variation,” “loss of appetite,” “psychological anxiety at baseline”). VEGF = vascular endothelial growth factor.

Regularization was conducted and a penalized regression model was established. Graphical summaries of the selection search are presented in **Figure 1A and B**, and parameter estimations of variable entry in the final step are summarized in **Table 3**. Three predictive variables [“HAMA Factor 1 at baseline (Psychological anxiety)”, “item 5 score of MADRS Scale (Loss of appetite)”, and “HAMD Factor 4 at baseline (Diurnal variation)”] were dropped out as meeting the cross-validation criterion and one variable (“Presence of psychotic symptoms”) was excluded by the researcher according to clinical experience.

**Table 3:** Parameter estimation of variables in the penalized regression model.

Variable entry	MLE estimate	Standard error	Pr > Chi-sq	Odds ratio	95% Wald CI		Tolerance value	*Cut-point
					Lower	Upper		
Baseline effect								
Age at onset (years)	−0.063	0.036	0.085	0.939	0.875	1.009	0.907	41.00
Presence of family history	4.559	1.525	0.003	95.50	4.812	>1000	0.919	1.000
IGF at baseline (ng/ml)	0.013	0.005	0.023	1.013	1.002	1.023	0.848	158.7
VEGF at baseline (pg/ml)	0.017	0.007	0.010	1.018	1.004	1.031	0.492	87.84
Delta effect								
Delta for FGF-2 (pg/ml)	−0.043	0.015	0.004	0.958	0.930	0.986	0.733	−8.170
Delta for NGF (pg/ml)	−0.187	0.075	0.012	0.829	0.716	0.960	0.548	1.411
Delta effect of HAMD Factor 2 (Weight loss)	−0.518	0.621	0.405	0.596	0.176	2.013	0.935	1.000

*Youden criterion.

**Figure 2:**
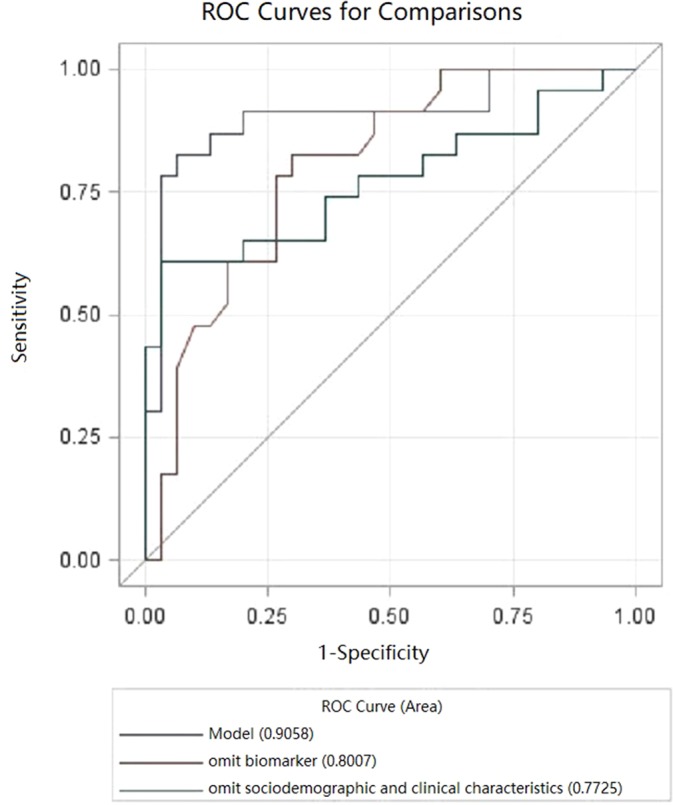
Receiver operating characteristic curve (ROC) curves for bipolar depressive disorder (BPD). Model, multivariate.

The final multiple diagnostic model using predictors combining neurotrophic biomarkers and clinical characteristics is shown in **Figure 2**, as well as biomarker predictor model alone and clinical characteristic predictor model alone. As seen in **Figure 2**, the multivariate model based on “Age at onset (years),” “Presence of family history,” “IGF at baseline (ng/ml),” “VEGF at baseline (pg/ml),” “delta for FGF-2 (pg/ml),” “delta for NGF (pg/ml),” and “delta effect of HAMD Factor 2 (Weight loss)” presented a good performance in detecting bipolar depressive disorder [Area Under Curve (AUC) = 0.9058, *P* < 0.05].

Models’ contrast estimation and testing results are also listed in **Table 4**. What can be clearly seen from the table is that the diagnostic model using biomarkers alone (AUC = 0.7725, *P* < 0.05) showed no significant difference with the diagnostic model using clinical characteristics (AUC = 0.8007, *P* < 0.05) in the level of consistency (*P* > 0.05).

**Table 4:** ROC association statistics and contrast estimation and testing results.

	ROC association statistics				Contrast estimation and testing**	
ROC Model	Area	Standard error	95% Wald CI		Estimate	Pr > Chi-sq
			Lower	Upper		
Model	0.9058	0.0472	0.8134	0.9982		
Model omit biomarkers	0.8007	0.0608	0.6816	0.9199	0.1333	0.0467
Model omit clinical characteristics’ effect	0.7725	0.0709	0.6336	0.9114	0.1051	0.0463
Model omit biomarkers vs. Model omit clinical characteristics’ effect					0.0283	0.7783*^+^

*Mann–Whitney; **Wald test.

^+^Model using biomarkers alone showed no significant difference with model using clinical characteristics alone.

In addition, “Presence of family history” and “Age at onset” in clinical data and “delta for FGF-2” in biomarker data showed a better predictive effect for the outcome.

## Discussion

This paper first explored the application of objective biological markers combined with clinical features in the field of psychiatric diagnosis. In this study, we found that peripheral neurotrophic factors had a stably good performance in identifying patients with bipolar disorder among depressive patients. Indeed, the distribution of neurotrophic factors had the same discriminatory power as clinical characteristics and could optimize prediction model performance as reliable indicators. This association persisted both pre-treatment and post-treatment.

The main findings in this study are consistent with those in previous clinical studies and multivariate biomarker discovery in mood disorder. Eleven variables including four for neurotrophic factors, two for demographic data, and five for symptomatic characteristics were identified as risk factors in discriminant analysis step. All 11 variables were considered as potential indicators of bipolar disorder that have been reported in other studies. In our study, these 11 variables together perfectly simulated the characteristics of bipolar disorder with a good fitting degree, while the false-positive rate reached up to 0.36%. After the penalized regression methods, clinical characteristics “Age at onset,” “Presence of family history,” and “delta effect of HAMD Factor 2 (Weight loss)” have frequently been identified as reliable distinguishable biomarkers for unipolar depression and bipolar depression. Similarly, the finding that higher distribution levels and trends of neurotrophic factors can effectively distinguish two types of mood disorders is consistent with those in our previous studies (10, 11).

There are also some inconsistencies between our study and other published studies. For example, variables that were considered as reliable discriminators in other studies failed to enter our final diagnostic model: “HAMD Factor 4 at baseline (Diurnal variation),” “item 5 score of MADRS Scale (Loss of appetite),” and “HAMA Factor 1 at baseline (Psychological anxiety).” In our preliminary feature selection by discriminant analysis, the three variables listed above were also identified as having a certain degree of discriminatory power. The major reason that they failed to enter the final model was that they did not meet the cross-validation criterion in the regularization step, which meant each of these variables would raise the model misclassification error rate. In the context of linear regression, cross-validation was a popular penalized regression method that enabled an assessment of the optimal complexity of a model and minimized the residual sum of squares by using a penalty on the size of the regression coefﬁcients so as to improve the overall prediction error (16). It was often exploited to decide a best-fit model that generally included only a subset of deemed truly informative features under the given data. But at the same time, this penalty might cause coefﬁcient estimates to be biased (in order to ensure cross-validation error), and that would remove some discriminatory variables out of the model if they lack enough effect size or potentially have a cross-correlation with other variables (17, 18). In summary, variables dropped in the regularization step not only help fit the statistical model but also lead to a higher risk of misclassification. That may also account for the inconsistency between different studies.

As mentioned above, the final model predictors included four baseline effects and three delta effects. All the predictors in the final model not only had adequate discrimination but also showed a stable and robust performance to reduce total misclassification error. Among them, “Presence of family history” and “delta for FGF-2” demonstrated both a univariate and a multivariate significant difference between the two groups and passed the regularization into the final model, showing their reliable and independent discriminant performance. Also, baseline effects “IGF-1” and “VEGF” that entered the stepwise discriminant model in the sixth and seventh steps showed a certain discernment. However, we should note that “VEGF” would slightly increase the cross-validation press of it and the tolerance was not high when entering the final regression model, as shown in **Table 3** and **Figure 1**. Moreover, “Age at onset” and “delta effect of HAMD Factor 2 (Weight loss)” were the other two predictions that show no statistical difference but pass the stepwise discriminant analysis as well as the penalized regression method. It was a matter of effect size versus statistical significance. As a general agreement (19, 20), effect size informed us about “the magnitude or practical importance of observed sample results” while statistical significance only evaluates the probability of obtaining the “Null hypothesis: A = B” outcome by chance. To be more specific, “Age at onset” held a good effect size but poor statistical significance. It helped to reduce the degree of overlapping between the two groups, but the differences in means were hard to be detected under the current sample size. According to **Table 3**, patients’ age at onset under 41 years old was more likely to be a bipolar one, and it was consistent with an earlier age at onset that had been published in many clinical studies (21). Notably, its 95% confidence interval of ratio was high and that might be the reason why it has not been detected. The same situation occurred in the variable “delta effect of HAMD Factor 2 (Weight loss)”. A depressed patient showing an improvement in weight loss was less likely to be a bipolar one (22). By adding this variable to the model, the Bayesian information criterion (SBC) would have a very small increase, but the Akaike information criterion (AIC) (23) would still decrease, which would still improve the model fit and cross-validation (**Figure 1**). This prompted us that, under the current sample, its convergence to the sample had reached its limit compared to other variables. In the small sample data feature selection, if only a univariate and unsupervised approach was applied, it was likely to be ignored. However, in the large sample of data, it might be easier to be identified and to obtain a good fit with better performance in different sample coherences. Larger samples were needed for further validation of the discriminant effect size of “Age at onset” and “delta effect of HAMD Factor 2 (Weight loss)”.

The current study further clarified our previous findings about the neurotrophic factor classification system in mood disorders and presented details regarding a high-dimensionality biomarker discovery in the clinical study (24). The search for biomarkers of psychiatric diseases was still in its infancy. In the absence of sufficient evidence, identification of only one or two types of biological signals might be a nontrivial task. Among published studies on biomarker detection, multivariate analysis was commonly used. For patients with mood disorders, there might be minor changes in the body’s multiple systems that was not individually significant. Biomarkers could only be identified by truly multivariate approaches (25, 26). However, the number of biomarkers selected should be less than seven times the number of observations. To kick out redundant information, there were common mistakes in many studies that controlled the number of variables to some notably identified ones by univariate tests (e.g., *t* test or *F* test). What’s worse, the penalized method has already had been applied to the dataset before feature and model selection. It would take a great risk of losing important discriminatory information and holding a strong univariate bias. An algorithm combining non-automatic data processing has its benefit but should avoid eliminating discriminatory information at the preprocessing step. Also, noise detection based on variable correlation analysis was neither efficient nor safe as it neglects the biological interpretation of biomarkers and the possibility of related variables’ cooperative discriminant power (25). In this sense, our algorithm nicely and efficiently circumvents this problem by adopting a supervised “wide in strict out strategy”. Since we exhaustively filtered variables of discrimination into the model and then strictly kicked out variables by combining automatic and non-automatic “punish” to an appropriate number under the current sample size, the results had considerable reliability and stability.

What makes our study different from other studies was that we separated original feature selection and classification system building. Many research designs often directly incorporated supervised/unsupervised algorithms into variable selection, such as using SVM, or principal component analysis, in order to screen for statistically significant variables. However, this algorithm was prone to loss of important discriminatory variables when it comes to proteins and gene analysis (22, 23), resulting in low research consistency when applied into the real world. This type of study neglects the importance of the biological interpretation of biomarkers and might completely drive the statistical analysis into a completely wrong discriminatory direction (26). Including all of the discriminatory information in the preliminary feature selection and then applying a supervised algorithm to boost the model performance may be a better alternative for biomarker detection. The intrinsic relationship might be muddier at this step, but stepwise analysis was listed vividly and was good for further analysis. As science cannot claim absolute truth, what we could approach was “tentative or approximate truth,” especially on psychiatry research that greatly relied on phenomenon-based diagnosis. By using supervised learning algorithms, we could be close to the biomarkers specific to bipolar disorder as much as possible. The model conducted an exhaustive search strategy and “Glmselect” in this paper may reflect some of the truth. It eﬀectively removed irrelevant and redundant features and was computationally efficient while showing detail. Glmselect was one of the easily conducted methods with the higher prediction accuracy and computational efﬁciency of penalized regression. There remained a wide variability in specific biomarkers that can distinguish bipolar depression from all depression; thus, simple and efficient screening tools that could be widely used in different samples should be widely applied.

Undoubtedly, there might be statistical weakness, since it was a small sample size data analysis. To solve it, we conducted a cross-validation and stepwise discrimination in the feature selection. In case of a small sample size, the use of a 10-fold cross-validation and sequential forward selection was confirmed to be a better choice than a simple wrapper (26). Also, ranking feature sets was often based on error estimation and regularization served to reduce the overfitting problem. Therefore, the sample size was appropriate to achieve reasonable precision in the validation.

Identification of bipolar disorder was a historically difficult problem. To date, there is no single biological indicator or classification system combining biological indicators that can distinguish bipolar depression from depression and that has a stable and specific good discernment (27). Just like looking for a needle in a haystack, we need a standard and efficient way to screen variables. Algorithms with carefully built-in feature selection often provide a better alternative. Not only should we focus on the screening of biomarkers, but we also need to establish a more standardized statistics strategy for clinical data. At the same time, neurotrophic factors of interest showed a good performance in comparison with clinical scales, deserving validated analysis in other larger samples.

## Ethics Statement

This study was carried out in accordance with the recommendations of Ethics Committee of Shanghai Mental Health Center with written informed consent from all subjects. All subjects gave written informed consent in accordance with the Declaration of Helsinki. The protocol was approved by the Ethics Committee of Shanghai Mental Health Center.

## Author Contributions

YZ performed the statistical analyses and wrote the manuscript. SH completed all of the data entry. ZL finished all of the laboratory work. YF, KJ, and SS were responsible for the diagnosis and clinical assessment of the participants. TZ provided assistance for the statistical analyses. XL designed and wrote the study protocol, managed the literature searches and analyses, and reviewed the manuscript. All authors approved the final version of this manuscript.

## Funding

This work was supported by projects from the Shanghai Health Bureau (2009098), the Natural Science Foundation of Shanghai (15ZR1435400), the National Natural Science Foundation of China (81000588), “Shanghai health system young talents training plan” in Shanghai Health Bureau (XYQ2011016), and the National Key Clinical Disciplines at Shanghai Mental Health Center (OMA-MH, 2011-873).

## Conflict of Interest Statement

The authors declare that the research was conducted in the absence of any commercial or financial relationships that could be construed as a potential conflict of interest.

## Supplementary Materials

The Supplementary Material for this article can be found online at: https://www.frontiersin.org/articles/10.3389/fpsyt.2019.00266/full#supplementary-material

Table S1 Sociodemographic and clinical characteristics of MDD patients and BPD patients.

Table S2 Scale scores and serum neurotrophic levels in MDD and BPD patients before and after treatment.
