# Supplementary material for: Detection Study of Bipolar Depression Through the Application of a Model-Based Algorithm in Terms of Clinical Feature and Peripheral Biomarkers
Source: Front Psychiatry. 2019 May 1;10:266. doi: 10.3389/fpsyt.2019.00266 (PMC6504694; doi:10.3389/fpsyt.2019.00266)
Supplement: Table S1 — Sociodemographic and clinical characteristics of MDD patients and BPD patients. [file Table_1.docx]

Supplementary Material

## Detection study of Bipolar Depression through the application of Model-based Algorithm in terms of clinical feature and peripheral biomarkers

Yanqun Zheng^1^, Shen He^1^, Tianhong Zhang^1,3^, Zhiguang Lin^2^, Shenxun Shi^4^, Yiru Fang^1^, Kaida Jiang^1^, Xiaohua Liu^1,3*^

**∗ Correspondence**

Xiaohua Liu

drliuxiaohua@gmail.com

**Supplementary Data**

The raw data for this study can be found in the *FigShare* <https://figshare.com/s/5f581c443e4b745f7b64>

**Supplementary Tables**

#### Table S1. Sociodemographic and clinical characteristics of MDD and BPD patients.

| Characteristics | Group1MDD | Group2BPD | Statistic | P value |
| --- | --- | --- | --- | --- |
|  | N=30 | N=23 |  |  |
| Age(years) | 47.17±12.50 | 44.74±15.49 | Z= -0.3054 | 0.7600 ^a^ |
| Gender male | 10(33.33%) | 10(43.48%) | χ^2^= 0.5703 | 0.4502 ^b^ |
| Education | | | | |
| Primary school and below | 13(43.33%) | 9(39.13%) | χ ^2^=2.9551 | 0.2282 ^b^ |
| High school | 10(33.33%) | 4(17.39%) |  |  |
| University and above | 7(23.33%) | 10(43.48%) |  |  |
| Marital Status | | | | |
| Married | 22(73.33%) | 15(65.22%) | χ ^2^= 0.7235 | 0.9051 ^c^ |
| Single | 4(13.33%) | 5(21.74%) |  |  |
| Divorced | 3(10.00%) | 2(8.70%) |  |  |
| Widow | 1(3.33%) | 1(4.35%) |  |  |
| Age at onset(years) | 40.23±11.97 | 33.61±13.09 | Z= -1.9033 | 0.0570 ^a^ |
| Duration of disorder(weeks) | 348.63±409.52 | 581.48±530.38 | Z=2.2559 | 0.0241 ^a＊^ |
| Duration of the present episode(weeks) | 14.50±19.73 | 15.09±12.34 | Z=1.0168 | 0.3092 ^a^ |
| Times of previous episode | 2.33±1.52;  2.00 (QR2.00)^M^ | 3.95±1.64;  3.50 (QR2.00)^M^ | Z=3.4131 | 0.0006 ^a＊^ |
| Presence of psychotic symptoms | 3(10%) | 0(0%) | χ ^2^= 2.4380 | 0.2489 ^c^ |
| First episode | 10(33.33%) | 1(4.35%) | χ ^2^= 6.6504 | 0.0149 ^c＊^ |
| Presence of family history | 2(6.67%) | 7(30.43%) | χ ^2^= 5.2170 | 0.0308 ^c＊^ |

^a^ Mann–Whitney U-test

^b^ Pearson χ ^2^

^c^ Fisher’s exact test

^＊^Significance difference (p<0.05)

^M^ Median (Interquartile Range)

**Table S2.** Scale scores and serum levels of neurotrophic factors in MDD and BPD patients before and after treatment.

|  | Group1MDD | Group2BPD | Statistic | P value |
| --- | --- | --- | --- | --- |
|  | N=30 | N=23 |  |  |
| item-1 score of HAMD Scale (Depressed mood) | | | | |
| baseline | 3.23±0.679 | 2.96±0.475 | Z=-1.729 | 0.084 ^a^ |
| delta for item-1 score of HAMD scale | 2.63±0.999 | 2.22±0.795 | Z=-1.600 | 0.110 ^a^ |
| item-2 score of HAMD Scale (Feeling of guilt) | | | | |
| baseline | 1.07±1.015 | 1.35±0.832 | Z=-1.746 | 0.081 ^a^ |
| delta for item-2 score of HAMD scale | 0.83±1.147 | 1.13±0.869 | Z=-1.644 | 0.100 ^a^ |
| item-3 score of HAMD Scale (Suicide) | | | | |
| baseline | 1.73±1.574 | 2.17±1.193 | Z=-1.182 | 0.237 ^a^ |
| delta for item-3 score of HAMD scale | 1.60±1.589 | 2.13±1.180 | Z=-1.439 | 0.150 ^a^ |
| item-4 score of HAMD Scale (Insomnia-Initial) | | | | |
| baseline | 1.53±0.571 | 1.26±0.752 | Z=-1.300 | 0.194 ^a^ |
| delta for item-4 score of HAMD scale | 1.30±0.750 | 0.96±0.825 | Z=-1.533 | 0.125 ^a^ |
| item-5 score of HAMD Scale (Insomnia-Middle) | | | | |
| baseline | 1.37±0.490 | 1.22±0.600 | Z=-0.857 | 0.392 ^a^ |
| delta for item-5 score of HAMD scale | 1.17±0.592 | 0.96±0.638 | Z=-1.217 | 0.224 ^a^ |
| item-6 score of HAMD Scale (Insomnia-Delayed) | | | | |
| baseline | 1.13±0.776 | 1±0.739 | Z=-0.654 | 0.513 ^a^ |
| delta for item-6 score of HAMD scale | 0.90±0.759 | 0.87±0.757 | Z=-0.144 | 0.885 ^a^ |
| item-7 score of HAMD Scale (Work and interests) | | | | |
| baseline | 2.60±0.932 | 2.52±0.593 | Z=-0.290 | 0.771 ^a^ |
| delta for item-7 score of HAMD scale | 2.07±1.285 | 2.00±0.905 | Z=-0.205 | 0.837 ^a^ |
| item-8 score of HAMD Scale (Retardation) | | | | |
| baseline | 1.37±0.999 | 1.74±0.964 | Z=-1.416 | 0.157 ^a^ |
| delta for item-8 score of HAMD scale | 1.10±0.995 | 1.26±1.176 | Z=-0.682 | 0.495 ^a^ |
| item-9 score of HAMD Scale (Agitation) | | | | |
| baseline | 0.53±1.008 | 0.70±0.765 | Z=-1.365 | 0.172 ^a^ |
| delta for item-9 score of HAMD scale | 0.43±1.006 | 0.52±0.730 | Z=-0.974 | 0.330 ^a^ |
| item-10 score of HAMD Scale (Anxiety-Psychic) | | | | |
| baseline | 1.67±.959 | 1.52±0.846 | Z=-0.681 | 0.496 ^a^ |
| delta for item-10 score of HAMD scale | 1.33±1.028 | 1.00±1.000 | Z=-1.027 | 0.304 ^a^ |
| item-11 score of HAMD Scale (Anxiety-Somatic) | | | | |
| baseline | 1.13±0.819 | 1.48±0.898 | Z=-1.361 | 0.173 ^a^ |
| delta for item-11 score of HAMD scale | 0.80±0.805 | 1.04±0.825 | Z=-1.375 | 0.169 ^a^ |
| item-12 score of HAMD Scale (Somatic symptoms Gastro-Intestinal) | | | | |
| baseline | 0.77±0.728 | 0.87±0.694 | Z=-0.566 | 0.571 ^a^ |
| delta for item-12 score of HAMD scale | 0.63±0.718 | 0.70±0.703 | Z=-0.206 | 0.837 ^a^ |
| item-13 score of HAMD Scale (Somatic symptoms General) | | | | |
| baseline | 0.70±0.651 | 0.91±0.733 | Z=-1.062 | 0.288 ^a^ |
| delta for item-13 score of HAMD scale | 0.40±0.724 | 0.57±0.728 | Z=-0.661 | 0.509 ^a^ |
| item-14 score of HAMD Scale (Genital symptoms) | | | | |
| baseline | 0.33±0.661 | 0.26±0.449 | Z=-0.012 | 0.990 ^a^ |
| delta for item-14 score of HAMD scale | 0.30±0.651 | 0.26±0.449 | Z=-0.295 | 0.768 ^a^ |
| item-15 score of HAMD Scale (Hypochondriasis) | | | | |
| baseline | 0.47±0.776 | 0.13±0.458 | Z=-1.878 | 0.060 ^a^ |
| delta for item-15 score of HAMD scale | 0.37±0.718 | 0.13±0.458 | Z=-1.401 | 0.161 ^a^ |
| item-16 score of HAMD Scale (Loss of weight) | | | | |
| baseline | 0.70±0.702 | 0.43±0.788 | Z=-1.683 | 0.092 ^a^ |
| delta for item-16 score of HAMD scale | 0.63±0.718 | 0.35±0.714 | Z=-1.771 | 0.077 ^a^ |
| item-17 score of HAMD Scale (Insight) | | | | |
| baseline | 0.83±0.747 | 0.87±0.694 | Z=-0.224 | 0.823 ^a^ |
| delta for item-17 score of HAMD scale | 0.40±0.675 | 0.39±0.583 | Z=-0.197 | 0.844 ^a^ |
| item-18 score of HAMD Scale (Diurnal variation) | | | | |
| baseline | 0.40±0.621 | 0.61±0.499 | Z=-1.662 | 0.096 ^a^ |
| delta for item-18 score of HAMD scale | 0.03±0.718 | 0.35±0.573 | Z=-2.149 | 0.032 ^a＊^ |
| item-19 score of HAMD Scale (Depersonalization/Derealization) | | | | |
| baseline | 0.07±0.254 | 0.09±0.288 | Z=-0.275 | 0.784 ^a^ |
| delta for item-19 score of HAMD scale | 0.03±0.183 | 0.04±0.209 | Z=-0.190 | 0.849 ^a^ |
| item-20 score of HAMD Scale (Paranoid Symptoms) | | | | |
| baseline | 0.27±0.691 | 0.04±0.209 | Z=-1.420 | 0.156 ^a^ |
| delta for item-20 score of HAMD scale | 0.17±0.648 | 0.04±0.209 | Z=-0.403 | 0.687 ^a^ |
| item-21 score of HAMD Scale (Obsessional/Compulsive Symptoms) | | | | |
| baseline | 0.23±0.626 | 0.04±0.209 | Z=-1.150 | 0.250 ^a^ |
| delta for item-21 score of HAMD scale | 0.13±0.571 | 0.00±0.000 | Z=-0.901 | 0.367 ^a^ |
| item-22 score of HAMD Scale (Sense of decline in ability) | | | | |
| baseline | 2.13±0.776 | 2.3±0.635 | Z=-1.145 | 0.252 ^a^ |
| delta for item-22 score of HAMD scale | 1.67±0.884 | 1.65±0.885 | Z=-0.048 | 0.962 ^a^ |
| item-23 score of HAMD Scale (Feeling of despair) | | | | |
| baseline | 2.13±0.973 | 2.17±0.717 | Z=-0.538 | 0.590 ^a^ |
| delta for item-23 score of HAMD scale | 1.73±1.048 | 1.61±0.839 | Z=-0.161 | 0.872 ^a^ |
| item-24 score of HAMD Scale (Feeling of inferiority) | | | | |
| baseline | 1.57±1.135 | 2.04±0.638 | Z=-1.837 | 0.066 ^a^ |
| delta for item-24 score of HAMD scale | 1.17±1.053 | 1.48±0.898 | Z=-1.279 | 0.201 ^a^ |
| HAMD-24 total score | | | | |
| baseline | 27.97±8.07 | 28.70±6.55 | Z=0.845 | 0.398 ^a^ |
| delta for HAMD-24 total score | 21.83±10.44 | 21.65±7.54 | Z=0.521 | 0.602 ^a^ |
| item-1 score of MADRS Scale (Apparent Sadness) | | | | |
| baseline | 4.07±1.230 | 3.83±0.887 | Z=-0.899 | 0.369 ^a^ |
| delta for item-1 score of MADRS scale | 3.40±1.567 | 2.91±1.240 | Z=-1.136 | 0.256 ^a^ |
| item-2 score of MADRS Scale (Reported Sadness) | | | | |
| baseline | 3.87±1.224 | 3.61±0.839 | Z=-0.825 | 0.409 ^a^ |
| delta for item-2 score of MADRS scale | 3.20±1.126 | 3.04±1.107 | Z=-0.198 | 0.843 ^a^ |
| item-3 score of MADRS Scale (Inner Tension) | | | | |
| baseline | 2.33±1.516 | 2.17±1.23 | Z=-0.407 | 0.684 ^a^ |
| delta for item-3 score of MADRS scale | 1.90±1.470 | 1.87±1.29 | Z=-0.186 | 0.852 ^a^ |
| item-4 score of MADRS Scale (Reduced Sleep) | | | | |
| baseline | 3.53±1.479 | 2.61±1.500 | F=2.242 | 0.029 ^b＊^ |
| delta for item-4 score of MADRS scale | 3.07±1.596 | 2.26±1.484 | Z=-1.819 | 0.069 ^a^ |
| item-5 score of MADRS Scale (Loss of appetite) | | | | |
| baseline | 2.60±1.404 | 1.96±1.224 | Z=-1.566 | 0.117 ^a^ |
| delta for item-5 score of MADRS scale | 2.37±1.273 | 1.83±1.230 | Z=-1.595 | 0.111 ^a^ |
| item-6 score of MADRS Scale (Concentration Difficulties) | | | | |
| baseline | 2.53±1.525 | 2.39±1.672 | Z=-0.348 | 0.728 ^a^ |
| delta for item-6 score of MADRS scale | 2.13±1.502 | 2.17±1.669 | Z=-0.165 | 0.869 ^a^ |
| item-7 score of MADRS Scale (Lassitude) | | | | |
| baseline | 3.07±1.388 | 2.96±1.186 | Z=-0.342 | 0.733 ^a^ |
| delta for item-7 score of MADRS scale | 2.67±1.626 | 2.30±1.550 | Z=-0.714 | 0.476 ^a^ |
| item-8 score of MADRS Scale (Inability to Feel) | | | | |
| baseline | 2.63±1.474 | 2.83±0.984 | Z=-0.529 | 0.597 ^a^ |
| delta for item-8 score of MADRS scale | 2.43±1.569 | 2.09±0.996 | Z=-0.913 | 0.361 ^a^ |
| item-9 score of MADRS Scale (Pessimistic Thoughts) | | | | |
| baseline | 3.43±1.357 | 3.3±1.329 | Z=-0.148 | 0.882 ^a^ |
| delta for item-9 score of MADRS scale | 3.13±1.548 | 3.00±1.348 | Z=-0.331 | 0.741 ^a^ |
| item-10 score of MADRS Scale (Suicidal Thoughts) | | | | |
| baseline | 2.57±2.128 | 2.74±2.005 | Z=-0.254 | 0.799 ^a^ |
| delta for item-10 score of MADRS scale | 2.50±2.193 | 2.70±1.964 | Z=-0.328 | 0.743 ^a^ |
| MADRS total score | | | | |
| baseline | 30.63±9.93 | 28.39±8.47 | F=1.37 | 0.390 ^b^ |
| delta for MADRS total score | 26.80±10.59 | 24.17±9.35 | F=1.28 | 0.351 ^b^ |
| item-1 score of HAMA Scale (Anxious mood) | | | | |
| baseline | 2.37±1.129 | 2.35±0.982 | Z=-0.150 | 0.881 ^a^ |
| delta for item-1 score of HAMA scale | 1.83±1.117 | 1.52±1.082 | Z=-0.689 | 0.491 ^a^ |
| item-2 score of HAMA Scale (Tension) | | | | |
| baseline | 1.37±1.098 | 1.87±1.14 | Z=-1.489 | 0.136 ^a^ |
| delta for item-2 score of HAMA scale | 1.10±.960 | 1.43±0.896 | Z=-1.480 | 0.139 ^a^ |
| item-3 score of HAMA Scale (Fears) | | | | |
| baseline | 1.17±1.117 | 1.22±1.043 | Z=-0.234 | 0.815 ^a^ |
| delta for item-3 score of HAMA scale | 0.97±1.066 | 0.96±0.825 | Z=-0.266 | 0.790 ^a^ |
| item-4 score of HAMA Scale (Insomnia) | | | | |
| baseline | 2.33±1.124 | 1.96±1.107 | Z=-1.290 | 0.197 ^a^ |
| delta for item-4 score of HAMA scale | 2.03±1.189 | 1.61±1.118 | Z=-1.452 | 0.147 ^a^ |
| item-5 score of HAMA Scale (Intellectual) | | | | |
| baseline | 1.37±1.066 | 1.61±1.118 | Z=-0.715 | 0.475 ^a^ |
| delta for item-5 score of HAMA scale | 1.07±1.048 | 1.22±1.204 | Z=-0.496 | 0.620 ^a^ |
| item-6 score of HAMA Scale (Depressed mood) | | | | |
| baseline | 3.03±0.669 | 2.96±0.562 | Z=-0.447 | 0.655 ^a^ |
| delta for item-6 score of HAMA scale | 2.47±0.860 | 2.22±0.850 | Z=-0.845 | 0.398 ^a^ |
| item-7 score of HAMA Scale (Somatic-muscular) | | | | |
| baseline | 1.13±1.074 | 1.61±1.234 | Z=-1.349 | 0.177 ^a^ |
| delta for item-7 score of HAMA scale | 0.90±1.094 | 1.17±0.984 | Z=-1.043 | 0.297 ^a^ |
| item-8 score of HAMA Scale (Somatic-sensory) | | | | |
| baseline | 1.00±.983 | 1.65±1.112 | Z=-2.045 | 0.041 ^a＊^ |
| delta for item-8 score of HAMA scale | 0.77±1.006 | 1.09±1.083 | Z=-1.211 | 0.226 ^a^ |
| item-9 score of HAMA Scale (Cardiovascular symptoms) | | | | |
| baseline | 0.77±0.898 | 0.78±0.998 | Z=-0.127 | 0.899 ^a^ |
| delta for item-9 score of HAMA scale | 0.67±0.844 | 0.65±0.982 | Z=-0.351 | 0.725 ^a^ |
| item-10 score of HAMA Scale (Respiratory symptoms) | | | | |
| baseline | 0.40±0.675 | 0.35±0.647 | Z=-0.307 | 0.759 ^a^ |
| delta for item-10 score of HAMA scale | 0.20±0.664 | 0.22±0.518 | Z=-0.234 | 0.815 ^a^ |
| item-11 score of HAMA Scale (Gastrointestinal symptoms) | | | | |
| baseline | 1.13±0.937 | 1.00±0.739 | Z=-0.517 | 0.605 ^a^ |
| delta for item-11 score of HAMA scale | 1.03±0.928 | 0.74±0.689 | Z=-1.074 | 0.283 ^a^ |
| item-12 score of HAMA Scale (Genitourinary symptoms) | | | | |
| baseline | 0.57±0.898 | 0.61±0.722 | Z=-0.611 | 0.541 ^a^ |
| delta for item-12 score of HAMA scale | 0.50±0.820 | 0.39±0.583 | Z=-0.140 | 0.889 ^a^ |
| item-13 score of HAMA Scale (Autonomic symptoms) | | | | |
| baseline | 0.73±0.828 | 1.13±0.869 | Z=-1.827 | 0.068 ^a^ |
| delta for item-13 score of HAMA scale | 0.60±0.855 | 0.74±0.752 | Z=-0.761 | 0.447 ^a^ |
| item-14 score of HAMA Scale (Behavior at interview) | | | | |
| baseline | 1.77±1.331 | 2.04±1.261 | Z=-0.792 | 0.428 ^a^ |
| delta for item-14 score of HAMA scale | 1.40±1.354 | 1.61±1.270 | Z=-0.608 | 0.543 ^a^ |
| HAMA total score | | | | |
| baseline | 19.13±7.34 | 21.13±9.44 | Z= 0.377 | 0.706 ^a^ |
| delta for HAMA total score | 15.53±7.98 | 15.57±8.68 | Z=-0.333 | 0.740 ^a^ |
| FGF-2 | | | | |
| baseline | 198.48 ±129.99 | 263.46±321.23 | Z=0.664 | 0.507 ^a^ |
| delta for FGF-2 | 10.32±22.17 | -7.58±69.74 | Z=-2.118 | 0.034 ^a＊^ |
| IGF-1 | | | | |
| baseline | 161.60±84.00 | 166.60±75.98 | F=1.22 | 0.822 ^b^ |
| delta for IGF-1 | 18.36±94.06 | -4.74±92.58 | F=1.03 | 0.377 ^b^ |
| VEGF | | | | |
| baseline | 98.08±57.14 | 174.2±223.30 | Z=0.817 | 0.414 ^a^ |
| delta for VEGF | -5.19±37.68 | -7.42±81.75 | Z=-0.942 | 0.346 ^a^ |
| NGF | | | | |
| baseline | 33.18±7.42 | 44.00±44.00 | Z=0.808 | 0.419 ^a^ |
| delta for NGF | 43.73±34.09 | 0.28±14.81 | Z=-0.835 | 0.404 ^a^ |

HAMD-24: 24-item Hamilton Depression Scale

MADRS: Montgomery Asperger Depression Scale

HAMA: Hamilton Anxiety Scale

^a^ Mann–Whitney U-test ^b^ Independent Samples t-test

^＊^Significance difference (p<0.05)
